# Catatonia in affective disorders

**DOI:** 10.1192/j.eurpsy.2026.10313

**Published:** 2026-07-31

**Authors:** M. Gietka

**Affiliations:** Masovian Viovodeship Hospital Drewnica, Ząbki, Poland

## Abstract

**Abstract:**

Catatonia is most commonly associated with schizophrenia and mood disorders, particularly depressive episodes; however, its occurrence during manic episodes of bipolar affective disorder remains relatively rare and insufficiently described. This presentation reports the case of a female patient with a long history of bipolar affective disorder and multiple psychiatric hospitalizations, who developed prominent catatonic symptoms during a severe manic episode.

During hospitalization, the patient exhibited marked psychomotor agitation, severely disorganized behavior, and repetitive motor automatisms, fulfilling criteria for an excited form of catatonia. The clinical course, diagnostic challenges, and therapeutic interventions are discussed in detail. The diagnostic work-up included laboratory testing, neuroimaging, and neurological hospitalisations to exclude organic causes of catatonic symptoms. Treatment strategies comprised pharmacotherapy tailored to both manic and catatonic symptoms, with careful monitoring of clinical response and adverse effects.

In addition, a brief review of the available literature on catatonia occurring in the course of mania is presented. Published reports suggest that such cases are uncommon and often underrecognized, contributing to diagnostic uncertainty and therapeutic difficulties. This case highlights the need for increased clinical awareness of catatonic manifestations in manic episodes and emphasizes the importance of early recognition to optimize treatment outcomes. Further research is warranted to establish clear diagnostic and therapeutic guidelines for this challenging clinical presentation.

**Disclosure of Interest:**

None Declared

